# Rapid screening for glucose-6-phosphate dehydrogenase deficiency and haemoglobin polymorphisms in Africa by a simple high-throughput SSOP-ELISA method

**DOI:** 10.1186/1475-2875-4-61

**Published:** 2005-12-15

**Authors:** Anders Enevold, Lasse S Vestergaard, John Lusingu, Chris J Drakeley, Martha M Lemnge, Thor G Theander, Ib C Bygbjerg, Michael Alifrangis

**Affiliations:** 1 Centre for Medical Parasitology, Institute for Medical Microbiology and Immunology, Panum, 24.2, Blegdamsvej 3, 2200 Copenhagen N, Denmark; 2 National Institute for Medical Research (NIMR), Box 4, Amani Medical Research Centre, Amani, Tanzania; 3 Joint Malaria Programme, Box 2228, Moshi, Tanzania

## Abstract

**Background:**

Mutations in the haemoglobin beta-globin *(HbB) *and glucose-6-phosphate dehydrogenase *(G6PD) *genes cause widespread human genetic disorders such as sickle cell diseases and G6PD deficiency. In sub-Saharan Africa, a few predominant polymorphic variants of each gene account for a majority of these deficiencies. Examining at a larger scale the clinical importance of these independent genetic disorders, their possible association with malaria pathogenesis and innate resistance, and their relevance for antimalarial drug treatment, would be easier if an accurate screening method with limited costs was available.

**Methods:**

A simple and rapid technique was developed to detect the most prominent single nucleotide polymorphisms (SNPs) in the *HbB *and *G6PD *genes. The method is able to detect the different haemoglobin polymorphisms A, S, C and E, as well as G6PD polymorphisms B, A and A- based on PCR-amplification followed by a hybridization step using sequence-specific oligonucleotide probes (SSOPs) specific for the SNP variants and quantified by ELISA.

**Results:**

The SSOP-ELISA method was found to be specific, and compared well to the commonly used PCR-RFLP technique. Identical results were obtained in 98% (haemoglobin) and 95% (G6PD) of the tested 90 field samples from a high-transmission area in Tanzania, which were used to validate the new technique.

**Conclusion:**

The simplicity and accuracy of the new methodology makes it suitable for application in settings where resources are limited. It would serve as a valuable tool for research purposes by monitoring genotype frequencies in relation to disease epidemiology.

## Background

Sickle cell disease and glucose-6-phosphate dehydrogenase (G6PD) deficiency are widespread inherited, but independent, human genetic disorders affecting millions of people [1-3]. Whereas G6PD deficiency is an x-linked enzymopathy and among the most common genetic disorder in humans [3], sickle cell disease is a common and severe haemoglobinopathy, caused by sickling haemoglobin [2]. The cytoplasmic G6PD enzyme is expressed in all tissues and is essential for the capacity of cells to withstand oxidative stress. Individuals with lowered levels of the G6PD enzyme are more susceptible to develop acute haemolytic anaemia [4]. Apart from childhood death, patients with sickle cell diseases suffer from diverse clinical conditions including anaemia, infarctions and other variable painful crisis throughout life [5].

The disorders arise from single nucleotide point mutations in the haemoglobin beta-globin (*HbB*) and glucose-6-phosphate dehydrogenase (*G6PD*) genes, resulting in single amino acid substitutions. Despite the fact that more than hundred allelic variants have been described for each polymorph gene-locus, only a few reach high frequencies and, thus, become of clinical importance [1,3].

In sub-Saharan Africa, G6PD is essentially a tri-allelic polymorphism. G6PD (B) is the most common allele with normal enzymatic activity; G6PD (A) is associated with a single amino acid substitution at codon (c) 126 where Asn is changed to Asp (N126D), causing around 85% of the normal enzymatic activity. The G6PD (A-) deficiency allele has a single amino acid substitution at c68 from Val to Met (V68M), always in conjunction with the N126D mutation [6]. The G6PD (A-) variant has only around 12% of normal enzymatic activity with frequencies of 5–25% of the affected population in sub-Saharan Africa [7]. Although most individuals with the G6PD (A-) polymorphic variant are asymptomatic, acute haemolytic anaemia can manifest in hetero- and homozyogte females as well as hemizygote males under oxidative stress of the red blood cells [7]. This condition can be induced by infections, anti-inflammatory agents and chemotherapeutics, including anti-malarials such as sulphadoxine-pyrimethamine, lapudrine-dapsone or primaquine, making it of importance to know the G6PD status of individuals receiving drug treatment [4].

The most prevalent polymorphic variants of the *HbB *gene consist of the normal gene (HbA) in which SNPs in the same amino acid position give rise to either the sickle haemoglobin S, (HbS, Glu to Val at c6 (E6V)) or the common west-African haemoglobin C (HbC, Glu to Lys at c6 (E6K)). A third SNP at position 26 creates the Southeast-Asian haemoglobin E (HbE, Glu to Lys (E26K)) variant, which is not found in Africa [1]. Apart from several other abnormal haemoglobin gene-combinations, such as beta-thalassaemia, sickle cell disease is found in individuals with homozygote HbSS (sickle cell anaemia) and heterozygote SC genotypes, whereas the inheritance of two C-alleles (HbCC) or one A plus one C or S allele (HbAS and HbAC) give rise to phenotypes associated with limited pathology [1]. Both haemoglobin S, C and E confer some degree of protection against severe malaria [8-10]. In sub-Saharan Africa, HbS gene frequencies exceed 25%, resulting in a widespread, but unequal distribution of the HbAS sickle cell trait [2].

The procedures for diagnosing sickle cell anaemia and G6PD deficiency includes the sickling test, haemoglobin electrophoresis [1,11] and enzymatic diagnostic assay [13]. Current approaches also deploy DNA-analysis using PCR-restriction fragment length polymorphism (RFLP) [9,14-16], allele-specific oligonucleotide hybridization [10,17] or dot-blot hybridization analysis of the appropriate PCR-amplified fragments [18]. However, these techniques can have difficulties in detecting heterozygotes and discerning false-negatives and false-positives thereby hampering specificity and sensitivity [19]. Furthermore, the methods are relatively laborious, technically demanding and expensive, and may, therefore, be inappropriate for screening large numbers of samples in resource-poor settings.

Here, a method for screening human populations for the different haemoglobin polymorphisms A, S, C and E as well as the G6PD polymorphisms B, A and A- by use of PCR and sequence-specific oligonucleotide probes (SSOPs) to detect the various SNPs and visualized in an ELISA system, is described and evaluated. The methodology is accurate and simple and would be applicable at laboratories with limited facilities.

## Methods

### Samples and DNA-extraction

Blood samples from 90 children under the age of five years, living in Mkokola village in North-Eastern Tanzania, collected on filter paper during a cross-sectional survey in March 2004, were used for validation of the method. Informed consent was obtained from parents or guardians of the study participants and the study protocol was ethically approved by the Tanzanian National Institute of Medical Research.

DNA samples of two donors with HbCC and HbAS genotypes and two donors with G6PD (A-), genotypes 68 MM (homozygote for 68 mutation) and G6PD (A), genotype 68VM/126ND (heterozygote for both mutations), respectively, served as positive controls. DNA extracted from volunteering Danish blood donors were used as controls for wildtype HbAA and G6PD (B) (68VV/126NN). Two samples from the villages Kwemasimba and Tamota in neighbouring areas were identified by the RFLP technique to be HbSS-type and HbAC-type, respectively, and were used as controls in comparison with the SSOP-ELISA technique. DNA was extracted from segments of bloodspots on filter paper in 96-well plate format by a Chelex-100 method described previously [20].

### PCR conditions

Primers, as described in Mombo et al. [16], were used to amplify a 919 bp fragment of the *G6PD *gene covering the two mutation sites at codon 68 and 126. The *HbB *gene was amplified by primers as described in Modiano et al. [9] to produce a 358 bp fragment covering the mutation sites at codon 6 and 26. Both reverse primers were biotinylated at the 5'-end by the supplier (MWG Biotech, Ebersberg, Germany).

One μl of DNA-extracted samples were amplified in 20 μl of PCR reaction mixture consisting of 0.2 mM of each dNTP, 2 μM of each primer-set, 1 unit of DNA Qiagen HotStart polymerase (Qiagen, Albertslund, Denmark), Qiagen buffer containing 2.5 mM MgCl_2_.

The PCR conditions for both the *G6PD *and *HbB *gene were 15 minutes of incubation at 94°C, followed by 45 amplification cycles of denaturation at 94°C for 30 seconds, annealing at 60°C for 30 seconds and elongation at 72°C for 90 seconds (60 seconds for the *HbB *gene), before the final elongation at 72°C for 10 minutes. The reactions were performed in 96-well PCR plates on a DNA thermal cycler (MJ Research, Albertslund, Denmark). A fraction of the PCR products were visualized by electrophoresis on a 1,5% agarose gel for 30 minutes for conformation of the amplification.

### Detection methods

#### SSOP-ELISA

Oligonucleotide-probes of 18 bases covering the mutation sites were designed and 3'-end conjugated with digoxigenin (MWG Biotech, Ebersberg, Germany) (Table 1). The detection of the SNPs were performed according to [21], with slight modifications. In brief, the amplified PCR products were diluted 1:2 in dH_2_0, denatured at 95°C for 5 minutes and kept at 4°C until use. 2 μl of the PCR products, conjugated with biotin, were added to streptavidin-coated (1 μg/ml PBS) ELISA plates (Maxisorp; Nunc, Roskilde, Denmark) containing 100 μl PBS with 0.05% Tween-20 and incubated for one hour at room temperature. The bound PCR-products were incubated with 8 nM of either probe in tetra-methyl ammonium chloride (TMAC; Sigma Aldrich, Seelze, Germany) solution (3 M TMAC, 50 mM Tris, pH 8.0, 0.1 sodium dodecyl sulfate, 2 mM EDTA, pH 8.0) at 53°C with shaking for an hour, followed by 2 × 10 minutes. TMAC-washing at temperatures set to 62°C for the haemoglobin probes and 65°C for the G6PD probes, respectively. The plates were incubated with peroxidase-conjugated anti-digoxigenin antibody (Roche Diagnostics, Mannheim, Germany) 1:1000 in PBS with 0.05% Tween-20 at room temperature for one hour, thereafter visualised by *o*-phenylene-diamine (OPD) (Dako, Glostrup, Denmark). The reaction was stopped with H_2_SO_4_, before measuring the optical density (OD) at 492 nm. Between each step, the ELISA plates were washed three times in PBS with 0.05% Tween-20. As the OD values of positive and negative controls changed between experiments, probably due to differences in strength of the probe binding, no fixed cut-off value could be specified; thus, for each SNP test, assay-specific cut-off values were defined.

**Table 1:** Sequences of the digoxigenin-conjugated sequence-specific oligonucleotide probes (SSOPs). The SSOP-ID named the sequences used in detection of the haemoglobin and G6PD single nucleotide polymorphisms (SNPs).

**SSOP ID**	**SSOP sequence**^1^	Nucleotide substitution	Amino acid substitution
*HbB*			
		
HbA-6:	5' TGACTCCTGAGGAGAAGT		
HbS-6:	5' TGACTCCTG**T**GGAGAAGT	A18T	E6V
HbC-6:	5' TGACTCCT**A**AGGAGAAGT	G17A	E6K
HbA-26	5' TTGGTGGTGAGGCCCTGG		
HbE-26:	5' TTGGTGGT**A**AGGCCCTGG	G77A	E26K
		
*G6PD*			
		
68V	5' CACCTTCATCGTGGGCTA		
68M	5' CACCTTCATC**A**TGGGCTA	G202A	V68M
126N	5' GCCACATGAATGCCCTCC		
126D	5' GCCACATG**G**ATGCCCTCC	A376G	N126D

^1^: Letters in bold indicate the nucleotide-substitutions

### Restriction fragment length polymorphism (RFLP)

In order to validate the method, comparative RFLP analyses were performed on similar PCR products used for the SSOP-ELISA technique. The 919 bp PCR fragment of the *G6PD *gene was digested with restriction enzyme NlaIII, which permits discrimination of the 68V and 68M genotypes [16], and BsmI, which allows discrimination of the 126N and 126D genotypes. The 358 bp fragment of the *HbB *gene was digested with restriction enzyme MnlI and DdeI for discrimination of the different haemoglobin polymorphisms, according to [9]. All digestions were carried out for a minimum of three hours, at 37°C for NlaIII, DdeI and MnlI, and 65°C for BsmI according to manufacturer's instructions, and resolved on 2–2.5% metaphor agarose gels (Medinova, Glostrup, Denmark).

## Results

### Specificity of the genotyping assay

In order to assess the capacity of the SSOP-ELISA technique to correctly detect HbB and G6PD SNPs, control DNA samples from individuals with known HbAA, HbCC, HbSS, HbAC and HbAS genotypes together with G6PD (68VV/126NN), (68VM/126ND) and (68MM/126DD) genotypes, were used to optimize the screening method.

**Figure 1:**
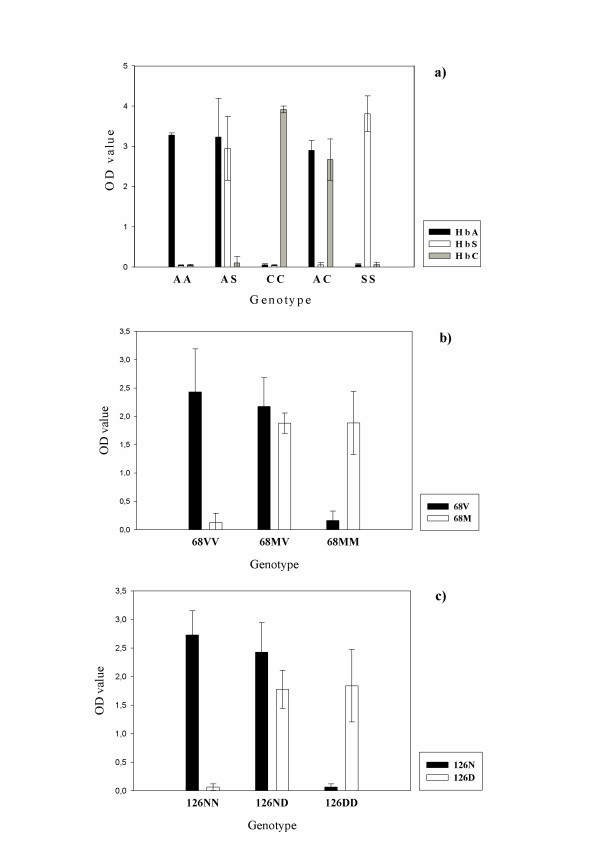
**Mean reactivity (OD value) and 95% confidence intervals in ELISA-SSOP assay of samples with known genotype. **Panel a) shows genotyping results of the *HbB *gene (genotypes AA, AS, CC, AC, SS) using probes reacting with HbA, HbS and HbC, respectively. Panel b) and c) shows genotyping results for G6PD for samples from individuals with the indicated genotypes using probes against 68V, 68M, 126N and 126D, respectively. All experiments were performed 4 times.

Using the control samples, all SSOPs identified with high specificity the appropriate SNPs in the DNA. The difference in OD-values between the cut-off value and the positive reactions (Δ-OD) was always higher than 2.0 for the haemoglobin samples and higher than 1.5 for the G6PD samples (Figure 1). A sample from an individual with the HbE mutation was not available, and therefore it was not possible to test the specificity of the HbE-26 probe. However, the probe HbA-26, specific for the normal genotype at c26, recognized the all samples with OD values above 2.0 (data not shown). The reaction intensity was equally strong in samples where each probe detected one allele (heterozygote and hemizygote) or two identical alleles (homozygote).

The procedure was optimized to different TMAC washing temperatures for the different probes, reaching an optimum specificity of 62°C and 65°C for HbB and G6PD probes, respectively. However, with slight decrease in specificity, both assays could be performed using a TMAC washing temperature in the range of 62–65°C. Although weak results could be obtained by applying the probes and PCR-products together at 53°C, saving one hour of the duration of the procedure, the strongest and most specific results were obtained by separating the PCR-product and probe incubations (one hour each). Similar, attempts to combine the two amplifications in one PCR-reaction (multiplex) also reduced the specificity and intensity of both PCR products. The cheaper and less hazardous tetramethylbenzidine (TMB) performed as well as OPD in the detection step.

### SSOP-ELISA versus the RFLP method

To test the SSOP-ELISA technique on samples from the field, DNA was extracted from filter paper blood spots collected from 90 children living in a Tanzanian village. The assay was able to screen these samples for the three haemoglobin and two G6PD SNPs during one day and determine the genotype frequencies (Table 2). The majority of the 90 samples were amplified by PCR, except three for the *HbB *gene and six samples for the *G6PD *gene. Of the PCR-amplified samples, the majority reacted with the SSOPs with a signal-to-background OD value above 1.0. As expected, no samples with HbC or HbE were identified in the village. Twelve individuals carried one HbS allele and had the HbAS genotype, corresponding to a prevalence of 14% of the sickle cell trait. The majority the individuals, 86% (75/87), carried the normal HbAA genotype.

**Table 2:** *HbB *and *G6PD *genotype frequencies for children living in a Tanzanian village and analysed by the SSOP-ELISA method.

**HbB Genotype**	**Numbers (87)**^a^	**G6PD Genotype**	**Numbers (84)**^a^	**68 Status**	**126 Status**	**Deduced Phenotype**^b^
		68–126				
AA	75 (86%)	VV-NN^c^	51 (61%)	Wildtype	Wildtype	G6PD B
SS	0	VV-DD^d^	8 (10%)	Wildtype	Mutant	G6PD A
AS	12 (14%)	M-D^e^	6 (7%)	Mutant	Mutant	G6PD A-
AC	0	VM-ND	10 (12%)	Single Mutant	Single Mutant	G6PD A
CC	0	VV-ND	8 (9%)	Wildtype	Single Mutant	G6PD A
SC	0	VM-DD	1 (1%)	Single Mutant	Mutant	G6PD A
No PCR-product	3	No PCR-product	6			

^a^: Numbers refer to the remaining individuals from the field with amplified PCR-product

^b^: Phenotype of the G6PD trait was not measured, but deduced based on the genotype

^c^: Out of the 51 individuals, 30 were males with the V-N genotype

^d^: Out of the 8 individuals, 5 were males with the V-D genotype

^e^: All 6 individuals were males hemizygote for M-D genotype

For G6PD, 61% (51/84) had the (68VV/126NN) normal genotype (G6PD B variant), whereas 20% (17/84) had the 68M mutation. These individuals could be divided into 6 (7%) with the deficient genotype (68M/126D – only hemizygote males), having the G6PD (A-) variant, and 11 (13%) which were heterozygote for the 68M mutation (10 with 68MV/126ND and 1 with 68MV/126DD) genotype having the G6PD (A) variant.

To verify the genotypes determined by the SSOP-ELISA assay, the PCR-products were processed by the RFLP method, which currently is commonly used for genotyping. For HbB, control and field samples gave identical results in 98% (88/90) of the cases. A minor disagreement was found in two samples typed as AS and AC, respectively. The samples had weak positive signals for the S and C probes close to the cut-off OD value of 0.3 in the SSOP-ELISA method. The restriction digestion analysis typed the samples as HbAA and not AC or AS.

Among 87 samples amplified for G6PD, there was 95% (83/87) and 97% (84/87) concordance between the two methods for the 68 and 126 mutation, respectively. Four samples with positive signals for 68V, showed weak signals with OD values between 0.2 and 0.5 for the 68M probe. Two of these were typed as 68MV by the RFLP method, whereas the two remaining were typed as 68VV. Another three samples, initially considered containing the 126D allele, typed as either 126DN or 126NN by the RFLP method. Ten samples (6 for the 68 mutation and 4 for the 126 mutation) with clear-cut OD values by the SSOP-ELISA method were difficult to interpret by the RFLP method due to ambiguous band-separation and band-resolution even after repeating the restriction digests. Comparisons of the results with the different methods are shown in Table 3.

**Table 3:** Comparison of results obtained with the SSOP-ELISA (horizontal) and RFLP (vertical). The single nucleotide polymorphisms (SNPs) in the *HbB *gene (top) and *G6PD *gene (bottom) were targeted using DNA extracted from control samples and 90 individuals from the field.

*HbB*	**SSOP-ELISA**						
**RFLP**	HbAA	HbAS	HbSS	HbAC	HbCC	No PCR prod.	
HbAA	74	1		1			
HbAS		11					
HbSS			1				
HbAC				1			
HbCC					1		
No PCR-prod.						3	

*G6PD*	**SSOP-ELISA**						
**RFLP**	68V	68M	68MV	126N	126D	126DN	No PCR prod.

68V	64		2				
68M		5					
68MV		2	8				
126N				50		1	
126D					14		
126ND					2	16	
No PCR-prod.							6
Inconclusive	4		2	2		2	

## Discussion

To allow large-scale screening of clinically important point mutations associated with G6PD deficiency and haemoglobinopathies, to be applied in resource-poor settings, a simple high-throughput SSOP-ELISA method was developed as an alternative to the conventional RFLP-method. Our new SSOP-ELISA method proved to be highly accurate in detecting the different SNPs of both the *HbB *and *G6PD *genes, with signal-to-background differences in OD values greater than 2.0 in most cases. Thus, with only a minimum discordance between the results obtained by the RFLP and SSOP-ELISA methods, the SSOP-ELISA technique confirmed to be an efficient alternative to the RFLP method. Although the SSOP-ELISA originally was developed for detecting *Plasmodium falciparum *haplotypes related to anti-malarial drug resistance, the technique has been shown to be adapted to analyse human genotypes as well. The results clearly showed that the OD values were not reduced in samples with two different SNPs a (heterozygote) compared to samples with two identical alleles (homozygotes). All individuals classified as heterozyogtes by the SSOP-ELISA were females, as expected.

The chosen PCR-products have the advantage of covering three important SNPs in the *HbB *gene and two important SNPs in the *G6PD *gene. The single PCR product from the G6PD gene covers both mutations, whereby amplification of two fragments, as has been done previously, is avoided [18,22]. The probes used were all designed to surround the SNP in question and only in the case of the 68M SNP, additional probes were designed to optimise a suitable binding reaction. In contrast, the PCR-RFLP methodology is dependent on recognisable restriction sites around the SNP, limiting the choice of size of the PCR fragment and binding of the primers. The SSOP-ELISA method also has the advantage of not relying on laborious electrophoresis work. Ambiguous results were more frequently observed with the RFLP method, caused by either incomplete enzymatic digestion of PCR fragments or inaccurate gel-resolution and band separation. The generation of ELISA-based OD values removes much of the conjecture over gel results, and a simple spreadsheet can be used for data analysis to identify samples, which need confirmation by the RFLP method. With duration of approximately 3 hours for the PCR amplification step followed by 4 hours for the SSOP-ELISA detection, 90 samples can easily be screened for both haemoglobin and G6PD genotypes or 180 samples for either haemoglobin or G6PD, during one day. Apart from being accurate and rapid, the technique is also relatively cheap as costs pr. sample analyzed and DNA extracted approximately is 1$.

From a practical point of view, setting up and running this new method only requires the use of relatively cheap equipment: a PCR thermal cycler, an incubation oven and an ELISA reader. These are increasingly becoming commonplace in many settings, also in laboratories in resource-poor settings in Africa. At this stage, the SSOP-ELISA technique has already been established at sites in Tanzania for the routine monitoring of molecular markers of *Plasmodium falciparum *drug resistance. The method can be adapted to other areas where different mutations predominate and even modified into a higher throughput system with access to more advanced and expensive equipment. In African populations, screening for the V68M mutant only will suffice, if the intention is solely to assess symptomatic G6PD deficiency [15,22].

The fact that this method is only able to detect specific SNPs, limits its use to monitoring already known genotypes, and sequencing analysis will be needed to identify unknown molecular markers or other variants of G6PD deficiency. Although by far the largest proportion of individuals with G6PD deficiency have the 68MM genotype, making it unlikely that a person in sub-Saharan Africa without the V68M mutation would be G6PD deficient [12], it is important to consider that ethnically different populations, e.g. with Arabic influence, could harbour other G6PD deficient variants (e.g. the Arabic G6PD^med ^variant) [12], and that rare deficiency mutations (G680T and T968C) could be present [7].

Limited access to and delayed diagnosis of sickle cell diseases and G6PD deficiency in tropical Africa may hamper prevention of severe illness and consequently lead to increases in morbidity and mortality [5]. Efficient high-throughput screening methods, as described here, may help to map the distribution of these inherited disorders and, thus, contribute as an epidemiological tool to characterize disease patterns. Molecular epidemiology of genes promoting or protecting against disease, including malaria, is important when large-scale interventions are planned, e.g. studies on malaria immunity, vaccine trials and drug trials.

## Conclusion

Here, a simple screening method is described, detecting, at molecular level and with limited costs, the most important human haemoglobin and G6PD polymorphisms associated with malaria. This assay represents a useful tool for analysing the relation between various combinations of these major genotypes and a variety of epidemiological factors in particular malaria immunity, incidence and severity. The method has proved to be accurate and rapid and will be beneficial at research institutions in resource-poor settings in Africa.

## Authors' contributions

AE conceived of the study, performed the experiments and wrote the manuscript. MA, CD, LV, JL, IB, ML and TT participated in manuscript preparation and design of the study. ML and JL supervised collection of samples. All authors read and approved the final manuscript.
